# Measuring Knowledge, Attitude, and Response (A-KAR) regarding chemotherapy among cancer patients: a cross-sectional study

**DOI:** 10.1038/s41598-026-62678-x

**Published:** 2026-07-20

**Authors:** Ahmed Reda Bahr, Basant Atef Ahmed, Hebatalla Abdelmaksoud Abdelmonsef Ahmed, Ibrahim A. Yakout, Mohamed K. Abdelhamid, Bassam Mohammed Khallaf, Ahmed Mohamed Musa Dana, Ahmed Hassan, Passant Ghoniem, Marwan Alqudah, Omar Hamdy

**Affiliations:** 1 Faculty of Medicine, Alexandria University, Alexandria, Egypt; 2 Faculty of Medicine, Tanta University, Tanta, Egypt; 3 Public Health & Community Medicine, Faculty of Medicine, Kafr-Elsheikh University, Kafr-Elsheikh, Egypt; 4 Faculty of Pharmacy, Zagazig University, Sharkia, Egypt; 5 Faculty of Medicine, Mansoura University, Mansoura, Egypt; 6 Oncology center, Faculty of Medicine, Mansoura University, Mansoura, Egypt; 7 Surgical Oncology Department, Oncology Center, Mansoura University, Mansoura, Egypt

**Keywords:** Chemotherapy, Cancer, Knowledge, Attitude, Response, Cross-sectional study, Cancer, Medical research, Oncology

## Abstract

Chemotherapeutic agents serve as a fundamental treatment approach for numerous cancer types. Providing patients with comprehensive information about chemotherapy’s purposes, advantages, disadvantages, and management strategies is crucial for decreasing anxiety, boosting treatment compliance, and improving general well-being. This study aims to measure the knowledge, attitude, and response **(**KAR) scores among cancer patients, in addition to identifying the potential associated factors for high scores. This observational cross-sectional study was conducted at the Oncology Center, Mansoura University, a tertiary care center, between September 2025 and October 2025. The study enrolled 669 cancer patients aged 18 years and above. Data were collected via a structured, Arabic-translated questionnaire developed from previous literature to assess KAR toward chemotherapy. Content validity was confirmed by three oncologists. Reliability and construct validity were evaluated using Cronbach’s alpha and exploratory factor analysis. Associated factors of total A-KAR scores were identified using univariate and multiple linear regression. A total of 669 patients participated with a mean age of 51.5 ± 13.2 years, predominantly female (68.2%). The mean knowledge score was 5.4 ± 2.3 out of 10, the mean attitude score was 10.8 ± 2.1 out of 15, and the mean response score was 2.7 ± 0.6 out of 3, with an overall total A-KAR score of 18.9 ± 3.9 (range: 5–28). Females exhibited significantly higher knowledge, attitude, and total A-KAR scores than males (*p* < 0.001). In the multiple regression model, higher A-KAR scores were independently predicted by female gender (β = − 0.329, *p* < 0.001), urban residency (β = 0.175, *p* = 0.037), absence of a family history of malignancy (β = − 0.164, *p* = 0.027), and receiving chemotherapy (β = − 0.478, *p* = 0.012). Duration since chemotherapy initiation was a strong independent associated factor, with significantly lower scores among those treated for less than one year compared with those treated for one to three years (all *p* < 0.001). Cancer patients demonstrated moderate overall knowledge and attitudes toward chemotherapy, with generally positive response behaviors. Several sociodemographic and clinical factors were significant independent associated factors of A-KAR scores. These findings highlight the need for targeted educational interventions that address knowledge gaps and support patients throughout their treatment journey.

**Supplementary Information:**

The online version contains supplementary material available at 10.1038/s41598-026-62678-x.

## Introduction

Chemotherapeutic agents serve as a fundamental and essential treatment approach for numerous cancer types worldwide. The widespread reliance on chemotherapy globally highlights its critical and irreplaceable role in comprehensive cancer care. During 2018, global statistics demonstrated that 57.7% of cancer patients (9.8 million out of 17 million diagnosed individuals) received chemotherapy treatment, underscoring its prevalence as a cornerstone therapy^1^. However, the inherent lack of selectivity of chemotherapy means that its effects on normal cells are inevitable, leading to the various and sometimes severe adverse reactions associated with these medications^2,3^.

The severe nature of chemotherapy’s side effects often leads to treatment non-compliance, worsened health status, decreased life quality, and sometimes fatal outcomes. These side effects vary depending on factors such as the type of drug used, dosage, frequency of administration, and whether chemotherapy is used in combination with other treatments^4,5^. Short-term side effects commonly associated with chemotherapy include nausea, vomiting, diarrhea, weight loss, infertility, early menopause, and mouth sores (stomatitis)^6^. Meanwhile, long-term side effects can include alopecia, myelosuppression, secondary cancers, anemia, neuropathy, and chronic fatigue^7^.

Providing patients with comprehensive information about chemotherapy’s purposes, possible adverse effects, and management strategies is crucial for decreasing anxiety, boosting treatment compliance, and improving general well-being^8^. Insufficient understanding can hinder proper chemotherapy administration, resulting in greater emotional suffering and reduced satisfaction. Educating patients about their prescribed medications and anticipated side effects should constitute a standard component of care provided by cancer treatment teams^9^. Research demonstrates that structured patient education interventions significantly improve knowledge regarding chemotherapy side effect management and enhance treatment compliance^10^.

Critical gaps in the literature are particularly evident as very few investigations have systematically characterized cancer patients’ knowledge, attitudes, and responses to chemotherapy^11,12^. These gaps are especially significant given unique contextual challenges, including health literacy variations, variable quality of provider explanations regarding side effects, inadequate patient preparation for symptom management, and cultural factors influencing health beliefs and treatment decisions. Healthcare systems in Egypt and similar lower-middle-income countries require foundational evidence regarding the baseline knowledge, attitudes, and coping responses of local cancer patient populations to inform the development and implementation of tailored, culturally appropriate educational interventions^13^.

This cross-sectional study addresses this evidence gap by systematically evaluating cancer patients’ knowledge, attitudes, and responses regarding chemotherapy at a single Egyptian center. By characterizing baseline understanding, this investigation will identify areas requiring enhanced patient education and provide evidence-based recommendations to optimize supportive care initiatives, ultimately improving treatment compliance, side-effect management, and patient quality of life.

## Study objectives and expected outcomes

### Primary objective

to assess the proper knowledge, attitude, and response (KAR) towards chemotherapy.

### Secondary objectives

1. To identify the sociodemographic associated factors of chemotherapy knowledge, attitude, and practice.
2. To validate a proper questionnaire assessing knowledge, attitude, and response to chemotherapy among cancer patients in the Arabic language, ensuring its further use among more cancer patients in countries whose residents speak Arabic, especially in the MENA region.

## Methodology

### Study design

This observational cross-sectional study was conducted at the Oncology Center, Mansoura University, a tertiary care center serving patients from Mansoura and the surrounding governorates, between September 2025 and October 2025.

### Study setting and population

The study was conducted at the Oncology Center of Mansoura University, a major tertiary care and referral center located in Dakahlia Governorate, Egypt, serving a large catchment area that includes Mansoura city and its surrounding regions in the Nile Delta. Eligibility criteria were established before the recruitment process. All adult patients (≥ 18 years) with a confirmed cancer diagnosis who were receiving care at the center, irrespective of chemotherapy status, were included.

### Sampling calculation

To recruit participants, convenience and snowball sampling methods were employed. The minimum sample size was calculated to ensure sufficient statistical power for detecting meaningful differences in KAR towards chemotherapy. With a 50% prevalence, a 5% margin of error, and a 95% confidence level, the minimum sample size requirement was 385 participants.

### Data collection tools and procedures

Data was collected using a well-structured questionnaire via face-to-face interviews with cancer patients at the Oncology Center of Mansoura University. The data was collected via an anonymous, and structured, in the Arabic language.

Lacking a proper clinical and valid questionnaire regarding KAR to chemotherapy, a careful review of the literature was conducted to develop an appropriate questionnaire assessing KAR to chemotherapy, based on an extensive literature review^14–17^.

The questionnaire consists of the following sections:

1. Sociodemographic sections included 12 questions regarding gender, marital status, education level, occupation, income, place of residence, cancer family history, chemotherapy duration, and cancer type.
2. The knowledge section consisted of 10 items. The score ranged from 0 to 10, comprising multiple-choice questions, with a score of 0 for a wrong choice or (don’t know) answer and a 1 for the correct option.
3. The attitude section consisted of 3 questions, with total scores ranging from 5 to 15, as there are 5 levels of answers for each question, thus giving 1, 2, 3, and 4 scores corresponding to strongly disagree, disagree, neutral, agree, and strongly agree, respectively.
4. Lastly, the patients’ response section, where participants were asked about their responses when experiencing any adverse effects from chemotherapy, assessed their health-seeking behavior. The item included four possible responses. Each response was scored on a four-point scale ranging from 0 to 3, reflecting the degree of positive health behavior as follows: Positive (3 points): I will go to the hospital, Partially positive (2 points): I will take the necessary medications at home, Neutral (1 point): I have not experienced any side effects. And Negative (0 points): I will not do anything.

**Scoring system**: the total Arabic version knowledge, attitude, and response (A-KAR) score ranged from 5 to 28 points. The data were scored using Bloom’s cut-off points: good if the score was between 80% and 100%, moderate if the score was between 60% and 79%, and poor if the score was less than 60%^18^.

### Translation procedure

To ensure the feasibility of conducting interviews with individuals whose mother tongue is Arabic, the questionnaire was forward-translated into Arabic by a professional translator and then backward-translated into English by an independent translator. To ensure an accurate translation, a panel of three oncologists reviewed the translation and then refined any minor discrepancies. The two versions of the questionnaire are depicted in Appendix 1.

### Content validity

Content validity of the questionnaire was assessed by a panel of three expert oncologists who served as expert reviewers. Each expert independently evaluated the relevance of every item using a 4-point ordinal scale, where 1 indicated not relevant and 4 indicated highly relevant. All items received ratings of either 3 or 4 from all reviewers, ascertaining a high Scale-Level Content Validity Index (S-CVI) of 100%.

### Ethical considerations

The Declaration of Helsinki’s guiding principles were followed during the study. The participants’ identities were preserved during the study, involving data collection and processing. The ethical approval was obtained from the Institutional Review Board (IRB) of the Faculty of Medicine, Mansoura University with ID number: **R.25.08.3328**.

### Statistical analysis

Data was coded, entered, and analyzed using the Statistical Package for the Social Sciences (SPSS) software, version 25.0. Descriptive statistics were calculated and presented as frequencies and percentages for categorical variables, and as means ± standard deviations (SD) for continuous variables. The normality of the data was tested using the Shapiro–Wilk test. The Mann–Whitney U test was used for comparisons between two groups, and the Kruskal–Wallis test was used for comparisons among more than two groups.

The reliability of the A-KAR Chemotherapy Questionnaire was assessed using Cronbach’s alpha coefficient, with values ≥ 0.70 considered acceptable^19^. The construct validity of the knowledge and attitude domains was examined using Exploratory Factor Analysis (EFA). Prior to doing EFA, the Kaiser–Meyer–Olkin (KMO) measure of sampling adequacy and Bartlett’s test of sphericity were performed. KMO statistics vary from 0 to 1, with values nearing 1 signifying greater appropriateness for factor analysis (KMO less than 0.6 denotes poor adequacy)^20^. If the p-value of the Bartlett test is below 0.05, factorial analysis is considered applicable^21^. The factors were found using Kaiser’s cutoff criterion with Eigenvalue (> 1) and a scree plot. For interpreting item loading, values of 0.32 are considered poor, 0.45 fair, 0.55 good, 0.63 very good, and 0.71 excellent^22^. Moreover, the Average Variance Extracted (AVE) was used to evaluate the amount of variance explained by each domain.

Spearman’s rank correlation coefficient was applied to examine the item-to-total correlation for each domain of the questionnaire and the associations among knowledge, attitude, and response domains.

To identify associated factors of the total A-KAR score, univariate and multiple linear regression analyses were conducted. Variables showing *p* < 0.20 in univariate analysis were entered into the multiple regression model using the enter method to control for potential confounders. The standardized regression coefficient (β) and corresponding *p*-values were reported. The *p-*value was established at less than 0.05.

## Results

### Sociodemographic characteristics

A total of 669 cancer patients were finally included. The participants’ mean age was 51.5 ± 13.2 years. Regarding the patients’ gender, females had the largest proportion of the gender distribution compared to males, 68.2% and 31.9% respectively. Most patients reside in rural areas (73.7%), while only 26.3% reside in urban areas. Married patients were the most (79.4%), followed by widowed (10.8%), then single (6.3%), and divorced patients represented the minority. About one-third (33.3%) were illiterate, accounting for the major proportion, followed by (26.8%) having secondary education, then (19.4%) having primary/preparatory education, and (18.8%) having a university education, while only (1.6%) had postgraduate education. Most patients were unemployed (76.3%), and only 23.6% were employed; 79.1% reported insufficient income. A family history of malignancy was present among 33.2% of participants. Most patients (81.9%) were currently receiving chemotherapy, with 38.9% having started treatment for 1–3 years. Table 1 shows the socio-demographic and clinical Characteristics.

**Table 1:** Socio-demographic and clinical training characteristics of the study participants (*N* = 669).

Studied variables		*N*	%
**Age (years)**	**mean ± SD (range)**	51.5 ± 13.2 (18.0–89.0)	
	**18–29**	40	6.0%
	**30–44**	148	22.1%
	**45–59**	278	41.6%
	**≥ 60**	203	30.3%
**Gender**	**Female**	456	68.2%
	**Male**	213	31.8%
**Residency**	**Rural**	493	73.7%
	**Urban**	176	26.3%
**Marital status**	**Married**	531	79.4%
	**Widowed**	72	10.8%
	**Divorced**	24	3.6%
	**Single**	42	6.3%
**Education**	**Illiterate**	223	33.3%
	**Primary/preparatory**	130	19.4%
	**Secondary**	179	26.8%
	**University**	126	18.8%
	**Post-Graduate**	11	1.6%
**Occupation**	**Employed**	158	23.6%
	**Not employed**	511	76.3%
**Income**	**Not enough**	529	79.1%
	**Enough**	124	18.5%
	**Enough and savings**	16	2.4%
**Family history of malignancy**	**Yes**	222	33.2%
	**No**	447	66.8%
**Chemotherapy intake**	**Yes**	548	81.9%
	**No**	121	18.1%
**Start of chemotherapy**	**1–6 months**	151	22.6%
	**7–12 months**	74	11.1%
	**1–3 years**	260	38.9%
	**> 3 years**	36	5.4%

### EFA and reliability analysis of the A-KAR

The Knowledge domain showed acceptable internal consistency (Cronbach’s α = 0.72), with item-total correlations ranging from 0.29 to 0.51 and factor loadings between 0.32 and 0.64. The AVE was 21.8%. The mean knowledge score was 5.4 ± 2.3 out of 10. The Attitude domain demonstrated a Cronbach’s α of 0.67, with moderate item-total correlations (0.46–0.52) and AVE of 40.7%. The mean attitude score was 10.8 ± 2.1 out of 15. Regarding the response domain, the majority of participants (82.8%) exhibited a positive response pattern, with a mean score of 2.7 ± 0.6 out of 3. The overall internal consistency for the full A-KAR scale was satisfactory (Cronbach’s α = 0.75), with a total mean score of 18.9 ± 3.9 out of 28. Table 2 presents the reliability and EFA of the A-KAR Chemotherapy Questionnaire.

**Table 2:** Reliability and EFA of the A-KAR chemotherapy questionnaire (*N* = 669).

A-KAR		*N* (%)	Item-Total Correlation	Cronbach’s α if Item Deleted	Factor loadings	Uniqueness
**Knowledge domain**	**K1**	468(70.0%)	0.37	0.70	0.44	0.80
	**K2**	251(37.5%)	0.31	0.71	0.37	0.86
	**K3**	432(64.6%)	0.32	0.71	0.36	0.86
	**K4**	442(66.1%)	0.29	0.72	0.32	0.89
	**K5**	197(29.4%)	0.51	0.68	0.64	0.59
	**K6**	114(17.0%)	0.44	0.69	0.53	0.71
	**K7**	552(82.5%)	0.31	0.71	0.36	0.86
	**K8**	289(43.2%)	0.49	0.68	0.58	0.65
	**K9**	315(47.1%)	0.45	0.69	0.55	0.69
	**K10**	574(85.8%)	0.34	0.71	0.37	0.85
	**Sum score** ***mean ± SD***	5.4 ± 2.3				
	**Cronbach’s α**	0.72				
	**AVE**	21.80%				
**Attitude domain**	**A1**	3.7 ± 0.9	0.46	0.60	0.60	0.65
	**A2**	3.5 ± 0.9	0.52	0.53	0.71	0.49
	**A3**	3.6 ± 0.9	0.46	0.60	0.60	0.64
	**Sum score** ***mean ± SD***	10.8 ± 2.1				
	**Cronbach’s α**	0.67				
	**AVE**	40.70%				
**Response domain**	**Positive**	554(82.8%)	0.13	0.76		
	**Partially positive**	45(6.7%)				
	**Neutral**	60(9.0%)				
	**Negative**	10(1.5%)				
	**Sum score** ***mean ± SD***	2.7 ± 0.6				
**Total A-KAR**	**Sum score** ***mean ± SD***	18.9 ± 3.9				
	**Cronbach’s α**	0.75				

AVE: Average Variance Extracted.

### Associated factors towards KAR

Knowledge scores significantly differed by age group (*p* = 0.003), with the highest mean among participants aged 30–44 years (6.0 ± 2.2). Females exhibited significantly higher knowledge, attitude, and total A-KAR scores than males (*p* < 0.001). Urban residents showed higher attitude (*p* < 0.001) and total A-KAR (*p* = 0.014) scores compared to rural participants. Participants with higher educational levels, particularly postgraduates, recorded significantly higher scores across knowledge, attitude, and total A-KAR domains (*p* < 0.001). Those without a family history of malignancy had significantly higher Knowledge (*p* < 0.001) and total A-KAR scores (*p* = 0.002). Participants currently receiving chemotherapy reported significantly better knowledge, attitude, response, and total A-KAR scores than those not receiving chemotherapy (all *p* < 0.001). The duration since starting chemotherapy was also a significant factor (*p* < 0.001), with the highest scores observed among those who had been on chemotherapy for 1–3 years. Table 3 presents the mean scores of the A-KAR domains according to socio-demographic and clinical characteristics.

**Fig. 1:**
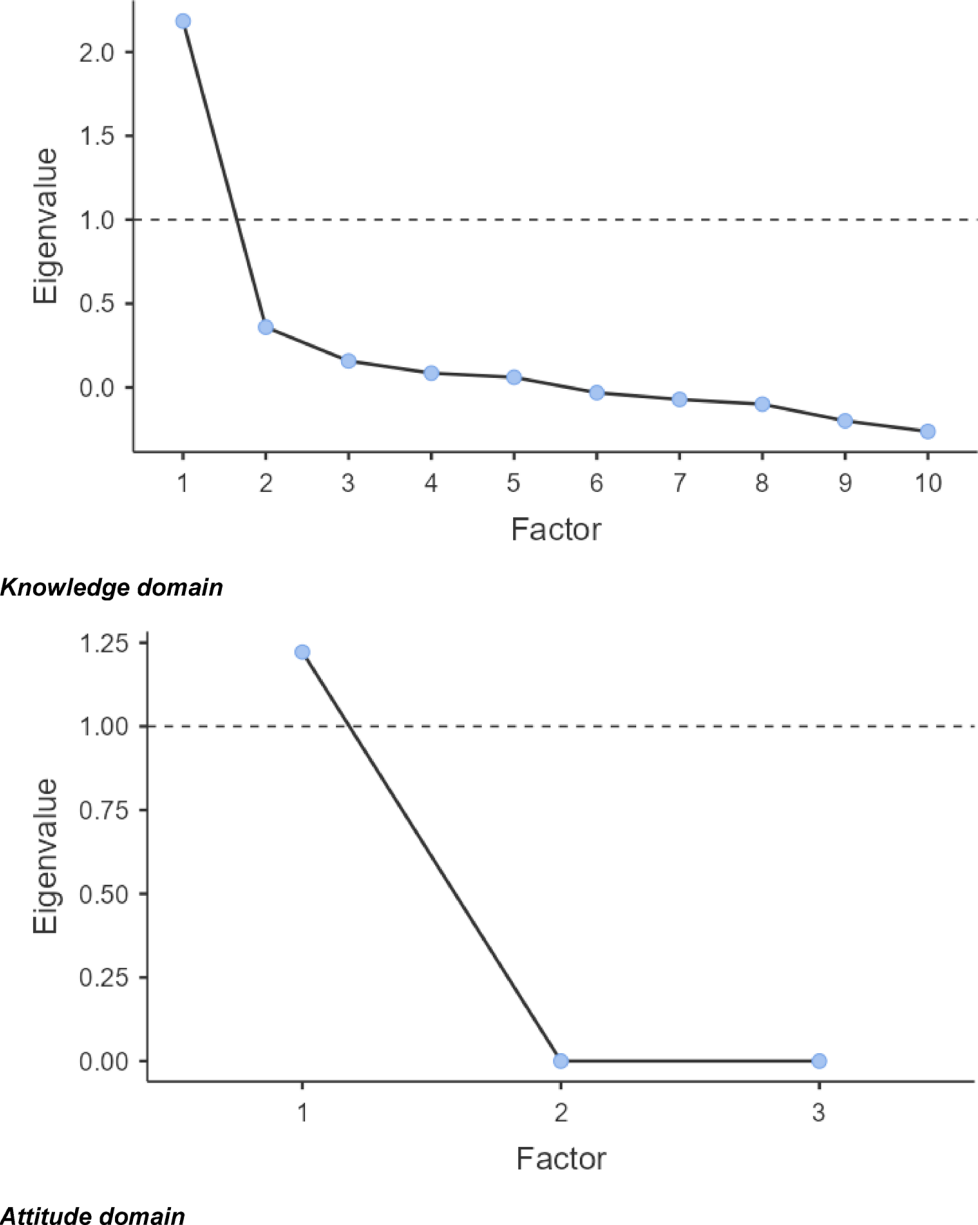
Scree plot of eigenvalues for the knowledge and attitude domains of the A-KAR chemotherapy questionnaire.

**Fig. 2:**
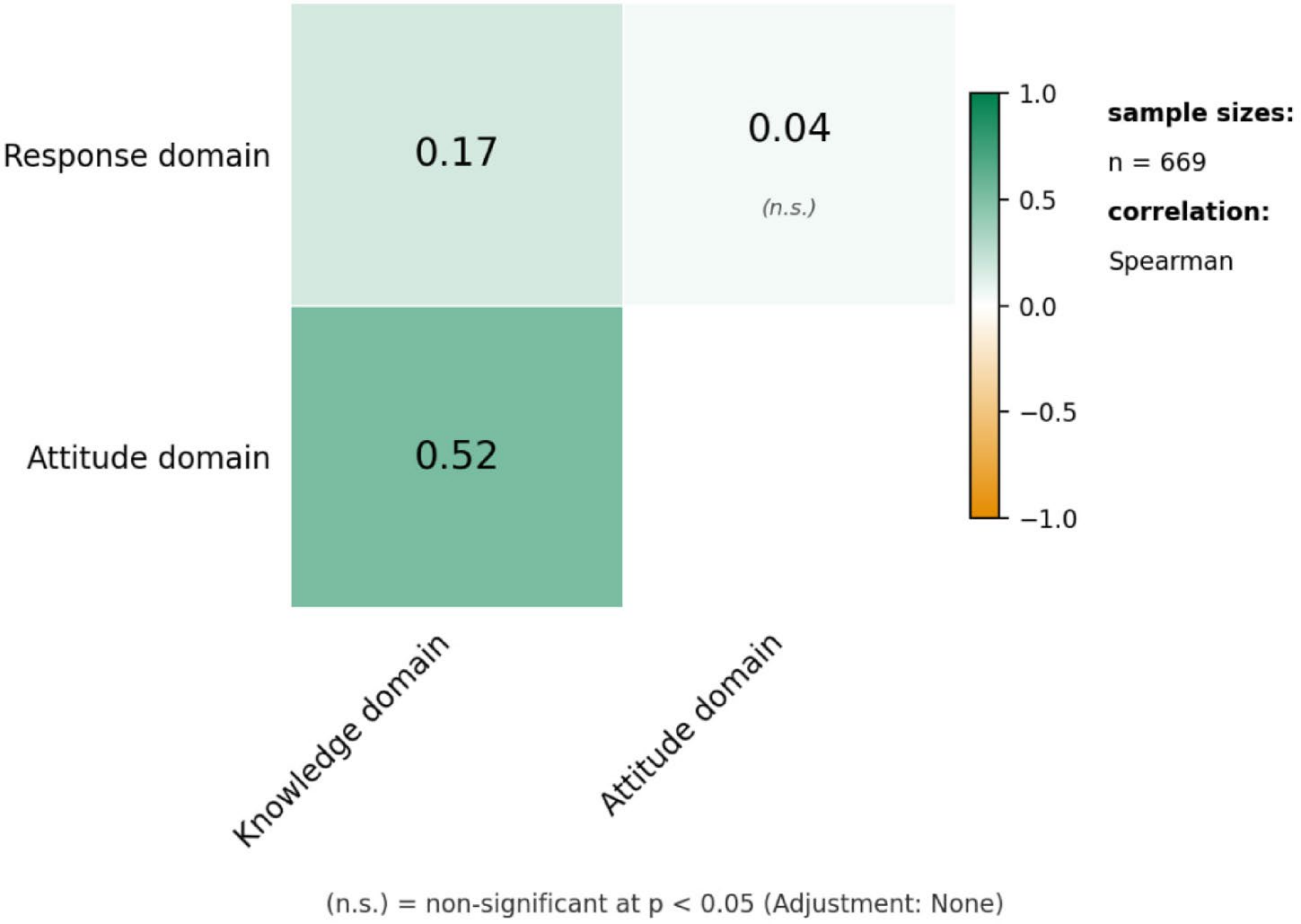
Spearman correlation matrix among knowledge, attitude, and response domains of the A-KAR chemotherapy questionnaire.

The scree plot illustrated in Fig. 1 confirmed the presence of one main factor corresponding to the knowledge and attitude domains. As shown in Fig. 2, Spearman correlation analysis revealed a significant positive correlation between the knowledge and attitude domains (rho = 0.52).

**Table 3:** Comparison of A-KAR chemotherapy questionnaire scores across socio-demographic and clinical training characteristics of the study participants (*N* = 669).

Studied variables		Knowledge domain	Attitude domain	Response domain	Total A-KAR
**Age (years)**	**18–29**	5.5 ± 2.3	11.0 ± 1.8	2.6 ± 0.8	19.1 ± 3.5
	**30–44**	6.0 ± 2.2	10.9 ± 2.2	2.7 ± 0.6	19.7 ± 4.0
	**45–59**	5.3 ± 2.3	10.9 ± 2.0	2.7 ± 0.7	18.9 ± 3.8
	**≥ 60**	5.1 ± 2.6	10.7 ± 2.1	2.7 ± 0.7	18.5 ± 4.3
	***P-value*** ^***a***^	0.003*	***0.555***	***0.712***	***0.061***
**Gender**	**Female**	5.7 ± 2.4	11.0 ± 2.1	2.7 ± 0.7	19.5 ± 4.0
	**Male**	4.9 ± 2.4	10.4 ± 2.0	2.7 ± 0.7	17.9 ± 3.8
	***P-value*** ^***b***^	< 0.001*	< 0.001*	***0.379***	< 0.001*
**Residency**	**Rural**	5.4 ± 2.4	10.7 ± 2.1	2.7 ± 0.7	18.8 ± 4.1
	**Urban**	5.6 ± 2.3	11.3 ± 2.0	2.7 ± 0.7	19.6 ± 3.7
	***P-value*** ^***b***^	***0.112***	< 0.001*	***0.668***	0.014*
**Marital status**	**Married**	5.5 ± 2.4	10.9 ± 2.0	2.7 ± 0.7	19.1 ± 3.9
	**Widowed**	5.0 ± 2.6	10.4 ± 2.3	2.6 ± 0.8	18.0 ± 4.6
	**Divorced**	4.2 ± 1.5	9.9 ± 2.3	2.6 ± 0.8	16.7 ± 3.2
	**Single**	6.0 ± 2.3	11.0 ± 1.7	2.8 ± 0.6	19.8 ± 3.6
	***P-value*** ^***a***^	***0.088***	***0.563***	***0.823***	***0.184***
**Education**	**Illiterate**	5.1 ± 2.4	10.8 ± 2.1	2.7 ± 0.7	18.7 ± 4.0
	**Primary/preparatory**	5.2 ± 2.5	10.3 ± 2.1	2.6 ± 0.7	18.1 ± 4.3
	**Secondary**	5.9 ± 2.4	11.0 ± 2.2	2.6 ± 0.7	19.5 ± 4.2
	**University**	5.3 ± 1.9	11.1 ± 1.7	2.8 ± 0.6	19.3 ± 3.0
	**Post-Graduate**	7.5 ± 2.4	12.0 ± 2.6	2.8 ± 0.6	22.4 ± 4.5
	***P-value*** ^***a***^	< 0.001*	0.001*	***0.187***	< 0.001*
**Occupation**	**Employed**	5.6 ± 2.4	10.8 ± 1.8	2.8 ± 0.6	19.2 ± 3.8
	**Not employed**	5.4 ± 2.4	10.8 ± 2.1	2.7 ± 0.7	18.9 ± 4.1
	***P-value*** ^***b***^	***0.382***	***0.964***	***0.795***	***0.515***
**Income**	**Not enough**	5.5 ± 2.5	10.9 ± 2.1	2.8 ± 0.6	19.1 ± 4.0
	**Enough**	5.2 ± 2.1	10.6 ± 2.1	2.5 ± 0.8	18.4 ± 3.9
	**Enough and savings**	6.1 ± 1.7	11.4 ± 1.5	2.4 ± 0.9	19.9 ± 3.2
	***P-value*** ^***a***^	***0.428***	***0.312***	< 0.001*	***0.287***
**Family history of malignancy**	**Yes**	4.9 ± 2.0	10.8 ± 2.1	2.7 ± 0.7	18.3 ± 3.5
	**No**	5.7 ± 2.5	10.9 ± 2.1	2.7 ± 0.7	19.3 ± 4.2
	***P-value*** ^***b***^	< 0.001*	***0.490***	***0.300***	0.002*
**Chemotherapy intake**	**Yes**	5.6 ± 2.4	11.0 ± 2.0	2.8 ± 0.6	19.4 ± 3.9
	**No**	4.7 ± 2.3	9.9 ± 2.0	2.5 ± 0.9	17.1 ± 4.0
	***P-value*** ^***b***^	< 0.001*	< 0.001*	< 0.001*	< 0.001*
**Start of chemotherapy**	**1–6 months**	4.2 ± 1.7	10.5 ± 1.9	2.8 ± 0.6	17.5 ± 2.9
	**7–12 months**	4.7 ± 2.0	10.6 ± 2.2	2.5 ± 0.9	17.8 ± 3.6
	**1–3 years**	6.8 ± 2.3	11.5 ± 1.8	2.8 ± 0.5	21.1 ± 3.7
	**> 3 years**	4.6 ± 1.6	10.8 ± 2.7	2.6 ± 0.8	18.1 ± 3.6
	***P-value*** ^***a***^	< 0.001*	< 0.001*	< 0.001*	< 0.001*

*Significant.; ^a^: Kruskal–Wallis test; ^b^: Mann-Whitney test.

**Fig. 3:**
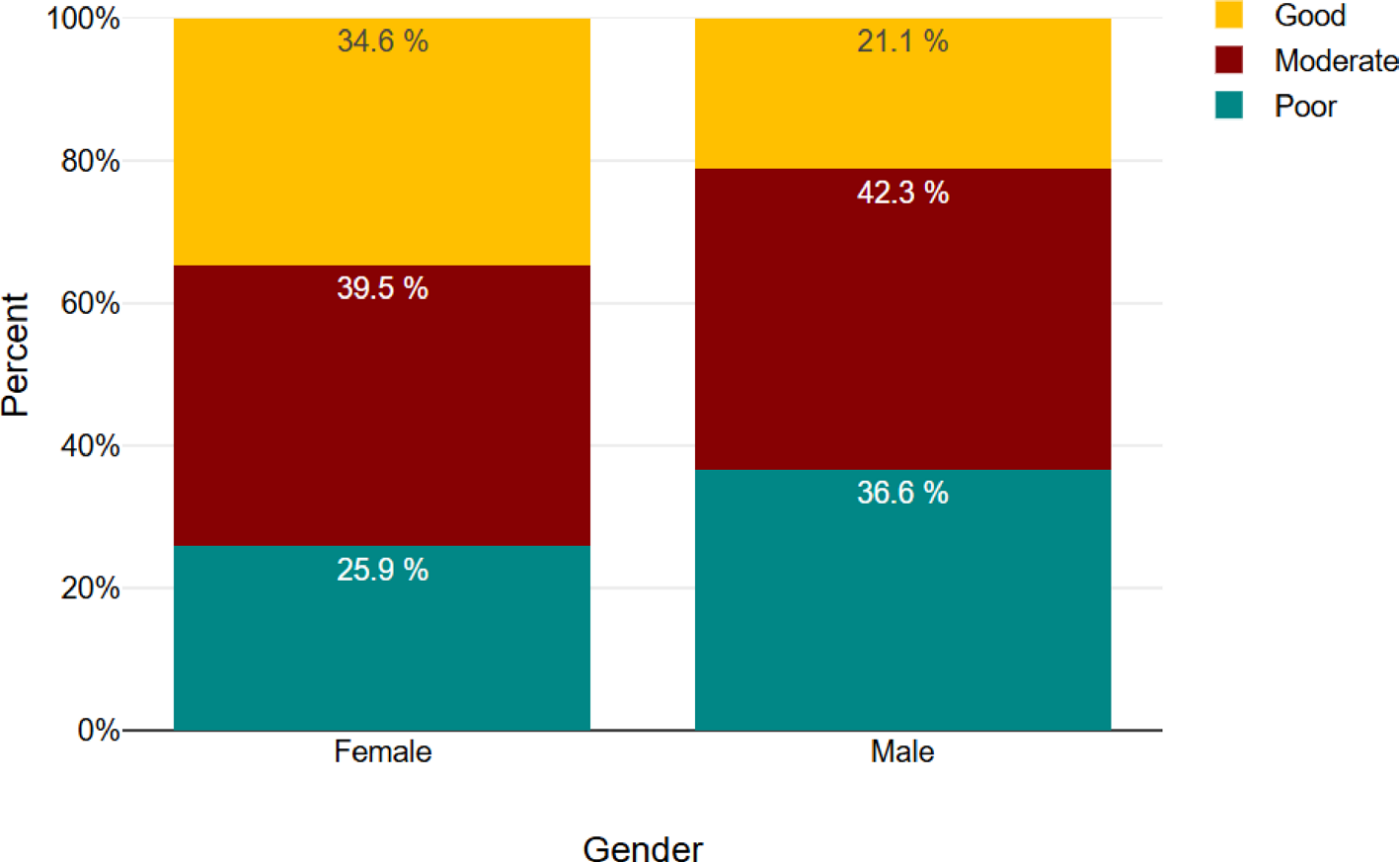
Comparison of total A-KAR score levels across gender.

Figure 3 illustrates the distribution of total A-KAR score levels across gender. Among females, 34.6% demonstrated a good A-KAR score, 39.5% showed a moderate score, and 25.9% had a poor score. In contrast, males exhibited a lower proportion of good scores (21.1%) and a slightly higher proportion of moderate scores (42.3%), while poor scores were most common among males (36.6%).

Univariate analysis identified several factors significantly associated with the total A-KAR score, including gender, residency, education, family history of malignancy, chemotherapy intake, and duration since starting chemotherapy. In the multiple regression model, males had lower scores than females (β = − 0.329, *p* < 0.001), Urban residency was independently associated with higher A-KAR scores (β = 0.175, *p* = 0.037). Participants with a family history of malignancy had significantly lower A-KAR scores compared with those without a family history (β = − 0.164, *p* = 0.027). Similarly, participants not receiving chemotherapy had significantly lower A-KAR scores than those receiving chemotherapy (β = − 0.478, *p* = 0.012). The duration since chemotherapy initiation also remained a strong independent factor, with significantly lower scores among those treated for less than one year compared with those treated for one to three years (all *p* < 0.001; Table 4).

**Table 4:** Univariate and multiple linear regression analysis of associated factors associated with the A-KAR Chemotherapy Questionnaire (*N* = 669).

Studied variables		Univariate		Multiple	
		β	*P*-value	Β	*P*-value
**Age (years)** ***Ref: 45–59***	**18–29**	0.041	0.807	−0.121	0.512
	**30–44**	0.189	0.062	0.008	0.930
	**≥ 60**	−0.095	0.298	−0.129	0.139
**Gender** ***Ref: female***	**Male**	-0.388	< 0.001*	-0.329	< 0.001*
**Residency** ***Ref: rural***	**Urban**	0.204	0.020*	0.175	0.037*
**Marital status** ***Ref: Ever married***	**Single**	0.224	0.161	0.228	0.190
**Education** ***Ref: illiterate***	**Primary/preparatory**	−0.150	0.170	−0.128	0.207
	**Secondary**	0.205	0.039*	0.083	0.418
	**University**	0.145	0.190	0.204	0.088
	**Post-Graduate**	0.917	0.003*	0.402	0.155
**Occupation** ***Ref: not employed***	**Employed**	0.071	0.439		
**Income** ***Ref: not enough***	**Enough**	−0.176	0.078	−0.102	0.270
	**Enough and savings**	0.198	0.435	0.299	0.191
**Family history of malignancy** ***Ref: no***	**Yes**	-0.234	0.004*	-0.164	0.027*
**Chemotherapy intake** ***Ref: yes***	**No**	-0.577	< 0.001*	-0.478	0.012*
**Start of chemotherapy** ***Ref: 1–3 years***	**1–6 months**	−0.909	< 0.001*	−0.884	< 0.001*
	**7–12 months**	−0.836	< 0.001*	−0.798	< 0.001*
	**> 3 years**	−0.771	< 0.001*	−0.711	< 0.001*

*Significant; β: standardized regression coefficient.

## Discussion

Healthcare workers in the field of oncology do not only fight cancer, but they also have to fight the misconceptions that patients have about their disease and their treatment. These misconceptions can lead patients to make wrong decisions about their care or cause a massive psychological load on patients who are already loaded. Before initiating therapy, the patient should understand the aim, advantages, drawbacks, and outcomes of their treatment plan. Every effort should be made to correct the knowledge and attitudes of cancer patients towards chemotherapy^14,23–26^. This study took a step forward on this long journey. It should be kept in mind that it is not a matter of “knowledge” only, but the attitude of the patient towards applying this “knowledge” matters as well. In almost all situations, the patient’s choice plays a principal role in the planning of the treatment pathway. Proper knowledge and a proper attitude are necessary for a successful decision^27,28^.

In this study, the mean attitude score (10.8 ± 2.1 out of 15) was higher than the mean knowledge and practice scores (5.4 ± 2.3 out of 10 & 2.7 ± 0.6 out of 3, respectively). This is consistent with a study, which exhibits a similar difference^14^. Another study also reported that knowledge scores about 50%^8^. This discrepancy highlights two points. The first is that while the patients may not have excellent knowledge about chemotherapy protocols, they can have better attitudes based on the trust in their treating physicians, who are considered the primary source of treatment-related information^28–30^. The second is that there is a need to carry out educational programs and support to improve this knowledge on a wide basis among patients, as well as the healthy population^29,30^.

One of the notable findings in our study is that knowledge scores significantly differed by age group (*p* = 0.003), with the highest mean among participants aged 30–44 years (6.0 ± 2.2). This does not cope with the previously published literature, where age was not a contributing factor to the level of knowledge^26^. Other studies evaluating the general knowledge of cancer showed heterogeneous results regarding the impact of age^31–33^. This may point out that age may affect chemotherapy knowledge. Therefore, further studies categorizing age into groups are warranted to demonstrate whether age affects chemotherapy knowledge or not.

In our study, females exhibited significantly higher knowledge, attitude, and total A-KAR scores than males (*p* < 0.001), which may be attributed to the cancer type, as 38.7% of patients had breast cancer, leading to the enrollment of more females. This was also not corroborated by other studies that showed no significant difference^14,26^. Yet other studies addressing general knowledge of cancer or cancer drugs showed similar results^34,35^. Therefore, other studies are needed to determine whether the knowledge score of females is attributed to cancer type or behavior.

Our study demonstrated three other notable findings that are relatively underexplored in literature. The first is that urban residents showed a higher attitude (*p* < 0.001) and total A-KAR scores (*p* = 0.014) compared to rural participants. This was not addressed in similar studies. The urban residents have better access to healthcare and information, which may justify how urban residents have higher A-KAR scores compared to rural residents. Yet another study showed familiar findings regarding the knowledge, attitudes, and practices of CML patients and their families toward TKI therapy^36^. The second key determinant was that the absence of a family history of malignancy was significantly associated with higher knowledge (*p* < 0.001) and total A-KAR scores. Other studies showed better knowledge of cancer risk factors in patients with a family history of cancer, whether cancer in general^37^ or oral cancer^38^. Patients with no family history of malignancy showed high scores of chemotherapy knowledge, suggesting they may have engaged more actively in information-seeking behaviors following diagnosis due to the novelty of the experience, whereas individuals with a family history may rely more on anecdotal beliefs or prior family experiences regarding chemotherapy, considering the possibility of residual confounding from unmeasured variables such as health literacy, psychosocial distress, caregiver involvement, and quality of physician counseling. The third is the duration since chemotherapy initiation, representing a strong independent associated factor, with significantly lower scores among those treated for less than one year compared with those treated for one to three years (all *p* < 0.001), indicating that chemotherapy knowledge strongly increases with chemotherapy duration, in addition to that in early durations may the impact of patient counseling is lower than patients treated over years.

Our study elicited that those with higher educational levels, particularly postgraduates, recorded significantly higher scores across knowledge, attitude, and total A-KAR domains (*p* < 0.001). This finding was also stated in another study, which showed significantly higher scores in the knowledge, attitude, and practice domains. Similarly, the study of Parker et al. highlighted the importance of education^26^.

### Strengths and limitations

This study revealed multiple important and new findings, starting with the use of a novel scale that explored the knowledge, attitude, and response among cancer patients. Regarding scale, we tested the scale reliability among cancer patients whose mother tongue was Arabic, in turn, opening doors for more longitudinal studies in the MENA region, and further use the scale worldwide by the English version. This study reported notable findings: knowledge scores significantly differed by age group, impact of gender on knowledge and total A_KAR scores shown as females exhibited significantly higher knowledge, attitude, and total A-KAR scores than males, impact of residency as urban residents showed a higher attitude (*p* < 0.001) and total A-KAR scores (*p* = 0.014) compared to rural participants, impact of family history as having a family history of malignancy was significantly associated with higher knowledge (*p* < 0.001) and total A-KAR scores, and impact of the duration of chemotherapy intake as patients started chemotherapy from less one-year showed lower scores compared to those who started chemotherapy from one to three years. These strengths were in addition to a large sample from a single cancer center, and conducting face-to-face interviews to ensure the participation of patients without access to the internet, and with low education. Further longitudinal studies in the MENA region will help us gain more understanding of the potential associated factors of higher A-KAR scores. Highlighting the need for national campaigns and education sessions for cancer patients for better awareness.

Our study has limitations: firstly, it is a single-center study, not guaranteeing a representative sampling across the country; however, the oncology center at Mansoura University represents a center that serves cancer patients in different regions with sociodemographic and socioeconomic disparities, in addition to being one of the largest public cancer centers in Egypt. Secondly, it provides data from patients treated in a public hospital, excluding those who were treated on a private or insurance basis, who may have different knowledge, attitudes, and practice patterns. Thirdly, the lack of correlation between these domains and their effect on patients’ oncological outcomes, if present. Additionally, using convenience and snowball sampling limits the generalizability of findings, and cross-sectional studies may have recall bias. In addition, lack of stratifying KAR scores according to cancer type and chemotherapy. This refers to several malignancy categories, and chemotherapy protocols had relatively small subgroup sizes, limiting statistical power and interpretability. Fifthly, the response domain remains limited by a single item, underscoring the necessity for more structure. Finally, EFA and reliability assessment demonstrated acceptable preliminary psychometric properties; both analyses were conducted using the same study population, which may limit the generalizability and stability of the factor structure. Additionally, the knowledge domain demonstrated relatively low average variance extracted (AVE), and the attitude domain showed borderline internal consistency reliability. Therefore, future multicenter studies using independent samples, split-sample validation approaches, and confirmatory factor analysis (CFA) are warranted to provide stronger evidence for construct validity, convergent validity, and reliability.

## Conclusion

This study highlights notable gaps in cancer patients’ KAR toward chemotherapy, despite generally positive response behaviors. The A-KAR Chemotherapy Questionnaire demonstrated good reliability and validity, allowing meaningful assessment of patient understanding. Higher A-KAR scores were associated with female gender, urban residence, absence of a family history of malignancy, and longer chemotherapy duration. These findings emphasize the need for structured, culturally tailored educational interventions within oncology centers in Egypt to improve patient knowledge, promote informed attitudes, and support appropriate health-seeking behaviors. Further research is recommended to evaluate targeted educational strategies using the validated A-KAR tool across broader and different populations.

## Supplementary Information

Below is the link to the electronic supplementary material.

Supplementary Material 1

## Data Availability

The Arabic and English versions of the questionnaire are in the supplementary material.

## Abbreviations

KAR: knowledge, attitude, and response
A-KAR: Arabic version of knowledge, attitude, and response
EFA: Exploratory Factor Analysis
KMO: Kaiser–Meyer–Olkin
AVE: Average Variance Extracted
