# Supplementary material for: Measuring Knowledge, Attitude, and Response (A-KAR) regarding chemotherapy among cancer patients: a cross-sectional study
Source: Sci Rep. 2026 Jul 20;16:22664. doi: 10.1038/s41598-026-62678-x (PMC13385768; doi:10.1038/s41598-026-62678-x)
Supplement: Supplementary file 1 — Supplementary Material 1 [file 41598_2026_62678_MOESM1_ESM.docx]

***Measuring Knowledge, Attitude and Response regarding chemotherapy among cancer patients; a single-center experience.***

***The English version***

**1.Socio-demographics**

1. **Age:**

● 18-24 years

● 25-31 years

● 32-38 years

● 39-45 years

● 46-50 years

● More than 50 years

1. **Gender**:

● Male

● Female

1. **Marital Status**

● Single

● Married

● Divorced

● Widow

1. **Educational status**

● Illiterate

● Primary

● Preparatory

● Secondary

● University

● Post-Graduate

1. **Employment status**

● Unemployed

● employed with part-time

● employed with full-time

● Retired

1. **Residency**

● Urban

● Rural

1. **Family income**

● Not enough

● Enough but not save money

● Enough and save money

1. **Do you have relatives diagnosed with cancer?**

● Yes

● No

1. **Their relationship?**

- From family (Father, mother, Son, daughter, husband, wife, uncle, etc…)
- Not from family (Friend, neighbor, work mate, etc…)
- I do not have relatives diagnosed with cancer

1. **When did you start chemotherapy?**

● 1-6 months

● 7-12 months

● 1-3 Years

● More than 3 years

1. **Cancer Type**

● Breast cancer

● Prostate cancer

● Colorectal cancer

● Gastric cancer

● Lung cancer

● Ovarian cancer

● Brain cancer

● Others

**2. Knowledge of Cancer Patients about chemotherapy**

1. **Do you think it was harmful when chemotherapy leaked out from the blood vessels to the surrounding tissues?**

● Yes

● No

● I don’t know

1. **What is your action you take when you have vomiting or during chemotherapy?**

● Eat as usual but with smaller amounts

● Avoid nasty smells, such as smoke flavor or perfume.

● Eat greasy food.

● Take a deep breath slowly, while you feel like vomiting.

1. **Do you think the hair loss from chemotherapy would be reversed?**

● Yes

● No

● I don’t know.

1. **What would you do if there is a drop in your white blood cells during chemotherapy?**

● Don’t contact a patient with a cold, flu, measles, or chicken pox patient.

● Get vaccinated for influenza and pneumonia.

● Play in public places.

● I don’t know.

1. **Which of the following phenomena will indicate low platelet levels?**

● Unexplained cyanosis of the skin

● Gums and nose bleeding

● Nausea

● I don’t know.

1. **What action would you take if your platelet level dropped during chemotherapy?**

● Brush your teeth with a soft bristle toothbrush

● Play basketball.

● Avoid injuries such as damage, burns.

● I don’t know.

1. **What would you do if you have an ulcer in the oral cavity during the therapy?**

● Eat hot food.

● Don’t eat warm and spicy food.

- Eat spicy food.

● I don’t know.

1. **What would you do if you noticed a change in your taste during chemotherapy?**

● Eat as usual but with smaller meals.

● Brush teeth or rinse mouth before meals

● Eat with metal tableware.

● I don’t know.

1. **What would you do if you suffered from constipation during chemotherapy?**

● Drink plenty of water.

● Take laxatives

● Lay on the bed to rest.

● I don’t know.

1. **Would you be required to drink more water during chemotherapy?**

- Yes
- No
- I don’t know.

3. **The Attitude of Patients Toward Chemotherapy Use**

1. **Most of the chemotherapy-induced side effects will disappear after the chemotherapy.**

- Strongly Agree
- Agree
- Neutral
- Disagree
- Strongly Disagree

1. **Chemotherapy-induced nausea and vomiting could be prevented or reduced.**

- Strongly Agree
- Agree
- Neutral
- Disagree
- Strongly Disagree

1. **The chemotherapy-induced adverse reactions could be reduced through self-care.**

- Strongly Agree
- Agree
- Neutral
- Disagree
- Strongly Disagree

4. **Patients’ response when they experienced any adverse effects from chemotherapy.**

I will go to the hospital.

I have not been exposed to any side effects.

I will not do anything.

I will take the necessary medications at home

***النسخة العربية***

1**- السمات الاجتماعية والديموغرافية**

1. العمر

• 18-24 سنة

• 25-31 سنة

• 32-38 سنة

• 39-45 سنة

• 46-50 سنة

• اعلي من 50 سنة

2. النوع

• ذكر

• انثي

3. الحالة الاجتماعية:

• أعزب

• متزوج/ة

• مطلق/ة

• أرمل/ة

4. المؤهل الدراسي:

• غير متعلم

• تعليم ابتدائي

• تعليم اعدادي

• تعليم ثانوي

• تعليم جامعي

• تعليم ما بعد الجامعي

5. الحالة الوظيفية

• غير موظف

• يعمل بـ دوام جزئي

• يعمل بـ دوام كلي

• متقاعد

6. الاقامة

• الحضر

• الريف

7. دخل الأسرة الشهري

• غير كاف

• كافي ولم ادخر

• كافي وأدخر

8. هل لديك اي من الاقارب مصابين بالسرطان؟

• نعم

• لا

9. صلة القرابة؟

• من العائلة (أب،أم،زوج/ة،ابن/ة،عم/ة، إلخ)

• ليس من العائلة (صديق/ة، جار/ة، زميل عمل، إلخ)

• ليس لدي أقارب مصابين بالسرطان

10. متي بدأت العلاج الكيماوي؟

• 6-1 اشهر

• 7-12 شهر

• 1-3 سنوات

• أكتر من 3 سنوات

11. نوع السرطان

• سرطان الثدي

• سرطان البروستاتا

• سرطان القولون/المستقيم

• سرطان المعدة

• سرطان الرئة

• سرطان المبيض

• سرطان الدماغ

• غير ذلك

**2- معرفة مرضي الاورام عن العلاج الكيماوي**

١. هل تعتقد انه مضر إن يتسرب العلاج الكيميائي من الاوعية الدموية الي الانسجة المحيطة؟

• نعم

• لا

• لا أعلم

٢. ما الدور الذي ستتأخذه إذا حدث لك غثيان او قيء خلال جرعة العلاج الكيميائي؟

• تناول كالمعتاد لكن وجبات اصغر

• الابتعاد عن الروائح النفاذة مثل رائحة السجائر او العطور

• تناول وجبات دهنية

• اخذ نفس عميق ببطء عند الشعور بالقيء

٣. هل تعتقد انه يمكن استعادة الشعر المتساقط من العلاج الكيميائي؟

• نعم

• لا

• لا اعلم

٤. ماذا ستفعل عند انخفاض خلايا الدم البيضاء خلال فترة العلاج الكيميائي؟

• تجنب الاختلاط بمريض مصاب بالبرد او الانفلونزا او الحصبة أو الجدري

• تناول اللقاح المخصص ضد الانفلونزا او الاتهاب الرئوي

• الذهاب للأماكن العامة

• لا اعلم

٥. أي من الظواهر الاتية تشير لانخفاض عدد الصفائح الدموية

• ظهور لون ازرق في الجلد غير مبرر

• حدوث نزيف من اللثة و الانف

• غثيان

• لا اعلم

٦. ماذا ستفعل إذا انخفضت عدد الصفائح الدموية خلال فترة العلاج الكيميائي؟

• غسل الاسنان بفرشاة ناعمة

• لعب كرة السلة

• تجنب الاصابات او الحروق

• لا اعلم

٧. ماذا ستفعل عند ظهور قرحة في الفم خلال فترة العلاج الكيميائي؟

• تناول اطعمة ساخنة

• عدم تناول اطعمة ساخنة او حارة

• تناول اطعمة حارة

• لا اعلم

٨. ماذا ستفعل إذا لاحظت تغير في المذاق فترة العلاج الكيميائي؟

• التناول كالمعتاد لكن بكميات اصغر

• غسل الاسنان او مضمضة الفم قبل الوجبات

• تناول الوجبات بأدوات مائدة معدنية

• لا اعلم

٩. ماذا ستفعل إذا عانيت من إمساك خلال فترة العلاج الكيميائي؟

• شرب كميات كبيرة من الماء

• تناول الملينات

• الاستلقاء علي السرير للراحة

• لا اعلم

١٠. هل مطالب أن أشرب ماء أكثر خلال فترة العلاج الكيميائي؟

• نعم

• لا

• لا اعلم

**3- سلوك مرضي الاورام حول استخدام العلاج الكيماوي**

1. معظم الاعراض الجانبية الناتجة عن العلاج الكيماوي ستختفي بعد انتهاء فترة العلاج

• اوافق بشدة

• اوافق

• متعادل

• لا اوافق

• لا اوافق بشدة

2. الغثيان او القيء الناتج من العلاج الكيماوي يمكن تجنبهم او تقليلهم

• اوافق بشدة

• اوافق

• متعادل

• لا اوافق

• لا اوافق بشدة

3. الاعراض الجانبية الناتجة عن العلاج الكيماوي يمكن تقليلها من خلال العناية الشخصية

• اوافق بشدة

• اوافق

• متعادل

• لا اوافق

• لا اوافق بشدة

4**- استجابة مرضي الاورام عند تجربتهم لأي من الاعراض الجانبية من العلاج الكيماوي**؟

• سأذهب للمستشفي

• لم اتعرض لأي اعراض جانبية سابقا

• لن افعل اي شيء

• سأتناول الادوية الضرورية في المنزل
